# A Sensitized Emission Based Calibration of FRET Efficiency for Probing the Architecture of Macromolecular Machines

**DOI:** 10.1007/s12195-013-0290-y

**Published:** 2013-07-11

**Authors:** Ajit Joglekar, Renjie Chen, Joshua Lawrimore

**Affiliations:** 1 Cell and Developmental Biology, University of Michigan Medical School, 109 Zina Pitcher Pl, 3067 BSRB, Ann Arbor, MI 48109 USA; 2 Department of Biology, University of North Carolina at Chapel Hill, Chapel Hill, NC USA

**Keywords:** Macromolecular architecture, FRET, Kinetochore

## Abstract

Macromolecular machines participate in almost every cell biological function. These machines can take the form of well-defined protein structures such as the kinetochore, or more loosely organized protein assemblies like the endocytic coat. The protein architecture of these machines—the arrangement of multiple copies of protein subunits at the nanoscale, is necessary for understanding their cell biological function and biophysical mechanism. Defining this architecture *in vivo* presents a major challenge. High density of protein molecules within macromolecular machines severely limits the effectiveness of super-resolution microscopy. However, this density is ideal for Forster Resonance Energy Transfer (FRET), which can determine the proximity between neighboring molecules. Here, we present a simple FRET quantitation scheme that calibrates a standard epifluorescence microscope for measuring donor–acceptor separations. This calibration can be used to deduce FRET efficiency fluorescence intensity measurements. This method will allow accurate determination of FRET efficiency over a wide range of values and FRET pair number. It will also allow dynamic FRET measurements with high spatiotemporal resolution under cell biological conditions. Although the poor maturation efficiency of genetically encoded fluorescent proteins presents a challenge, we show that its effects can be alleviated. To demonstrate this methodology, we probe the *in vivo* architecture of the γ-Tubulin Ring. Our technique can be applied to study the architecture and dynamics of a wide range of macromolecular machines.

**Electronic supplementary material:**

The online version of this article (doi:10.1007/s12195-013-0290-y) contains supplementary material, which is available to authorized users.

## Introduction

The nanoscale protein architecture of macromolecular complexes is essential for their function and regulation. For example, architecture is critical for functional and regulatory mechanisms of the kinetochore, a macromolecular machine that drives the movement and accurate segregation of chromosomes during cell division.^28^ The kinetochore uses multiple copies of at least eight different protein complexes to couple chromosome movement to the plus-end of a growing and shrinking microtubule (MT).^15^ This protein machinery also encodes a signal transduction system to monitor whether the kinetochore is attached to a MT, and to communicate this information to the biochemical cascade that controls the cell cycle. The nanoscale architecture of kinetochore subunits relative to the MT tip is an essential determinant of the molecular mechanism of both these functions.^4^ Multi-protein assemblies are also ubiquitous in the cell. For example, the endocytic protein coat incorporates many different proteins in multiple copies to achieve its function. The spatiotemporal sequence of recruitment and action of the endocytic coat factors dictates their functional and regulatory mechanisms.^17^ The changing nanoscale organization endocytic proteins can illuminate the functional and regulatory mechanisms that enable efficient coat maturation.^19^ Thus, the definition of the nanoscale organization of macromolecular machines is an important step in defining their function and regulation.

Despite the significance of protein architecture to macromolecular machines, the inherent complexity of such machines presents a significant hurdle. Biochemical purification or reconstitution or structural characterization of machinery containing a large number of subunits can be a challenging proposition. Furthermore, knowledge of the *in vivo* architecture is necessary to corroborate the success of the *in vitro* results. Progress in super-resolution microscopy provides a highly promising approach to obtaining *in vivo* architecture. However, the complexity of macromolecular structures also limits the effectiveness of super-resolution microscopy techniques.^11^ For example, the kinetochore uses ~40 molecules of at least 3 MT-binding proteins which all localize within a 40–60 nm longitudinal span along the MT axis and around the 25 nm diameter MT circumference. Resolving the positions of individual molecules within the kinetochore requires a resolution of <10 nm in 3D, which is beyond the capabilities of even the most advanced super-resolution microscopy techniques especially inside live cells. Therefore, alternative approaches are necessary to reconstruct macromolecular architecture and to detect any dynamic changes within the architecture.

FRET is an effective technique for probing macromolecular architecture.^10^ FRET can be used to measure the average proximity of adjacent molecules within the macromolecular machine. The architecture can then be reconstructed from pairwise measurements of protein proximities within the macromolecular machine.^24^,^25^ To convert protein proximities to physical separation, FRET efficiency must be determined.^8^ However, FRET efficiency measurement *in vivo* can be technically challenging. Fluorescence Lifetime Imaging (FLIM), which is a widely used technique to measure FRET efficiency directly, requires specialized instrumentation. In addition, accurate determination of fluorescence lifetime requires the detection of a large number of photons. This can be challenging if the macromolecular structure being studied incorporates a small and stable number of fluorophores, or if FRET efficiency being measured is low. Acceptor photobleaching is another commonly used method. It uses the ratio of donor intensity before and after photobleaching acceptor molecules to measure FRET efficiency. Due to irreversible photobleaching of the acceptors, this method provides only one measurement of FRET efficiency for a given structure preventing dynamic FRET measurement.^27^ Acceptor photobleaching method is also difficult to implement if the structure being studied is dynamic, or if there is a constant turn-over of proteins within the structure. To overcome these challenges, we present a methodology based on fluorescence intensity measurements conducted on a standard epifluorescence microscope.

## Materials and Methods

### Strain Construction

Haploid budding yeast strains were constructed through PCR-based transformation of wild-type strains using standard methods.^16^ We used GFP(S65T) as the donor fluorophore and mCherry as the acceptor. Both were fused to the carboxyl terminus of selected proteins using a seven amino acid linker with the sequence ‘RIPGLIN’. Cells were grown in YPD (or YP Raffinose + Galactose) media at 25 °C, and imaged at room temperature (~25 °C). For imaging, mid-log phase cells were rinsed and concentrated in synthetic media supplemented with essential amino acids. Cells were immobilized on ConA coated coverslips and sealed with VALAP to prevent evaporation. Imaging experiments lasted <30 min.

### Microscope Set Up

Fluorescence imaging was conducted on a Nikon Ti-E inverted microscope with a 1.4 NA, 100×, oil immersion objective. The microscope was equipped with XY linear encoders and a piezoelectric Z-stage (Prior). Excitation was achieved with the Lumencor LED light engine (472/20 nm for GFP and 543/20 nm for mCherry). Excitation light was filtered with a dual-band excitation filter ET/GFP–mCherry (59002x) and dichroic (89019bs), both from Chroma. Emission light was collected from the bottom port of the microscope and passed through the Dual-View attachment to simultaneously acquire GFP and mCherry emission. The Dual-view slider contained the emission-side dichroic (T560lpxr) and two emission filters: ET525/50m for GFP and ET595/50m for mCherry. Images were acquired with an Andor iXon camera (Andor Technology) with a pixel size of 160 nm. The camera was operated in the conventional mode.

### Imaging Protocol

The cells were selected and manually moved to the central ~16 *μ*m^2^ of the field of view using transmitted light. Limiting image analysis over this region ensured nearly uniform excitation intensity for all the cells measured. A Z-stack of images was obtained with 200 nm spacing between successive images. The *Z*-axis fluorescence distribution for metaphase and anaphase clusters can be approximated with a Gaussian function, and the 200 nm spacing ensures that the maxima of this distribution (the in-focus plane) is under-estimated by only ~5% on average.^16^ At each Z position, the first image obtained was with mCherry excitation to measure acceptor fluorescence, and then with GFP excitation. Emission-side beam-splitter allowed us to acquire both GFP and FRET images simultaneously. We used 100 ms integration time for both GFP and mCherry in most experiments (200 ms for competitive recruitment of Nuf2-GFP/Nuf2-mCherry). The excitation intensity for both GFP and mCherry was adjusted so as to achieve a sufficiently high signal to noise ratio, especially in the GFP and FRET channels while keeping relatively low integration time. We also ensured that the excitation intensity used in our experiments fell in the linear response region of both fluorophores. Therefore, the integration time and/or excitation intensity can be proportionally increased in both channels if necessary. Photobleaching during image acquisition was minimal under these conditions.

### Image Analysis

Image analysis was conducted in a semi-automated graphical user interface written in MatLab. The GUI allows the user to click close to a metaphase or anaphase kinetochore cluster (visually identified from the separation between the two kinetochore clusters within the cell). The program locates the in-focus plane by searching for the maximum intensity pixel in 3D in a set vicinity of the click. For metaphase cells, the GUI selects a 6 × 6 pixel region positioned to maximize the cumulative intensity of the central 4 × 4 region. This region of interest selection was chosen because of the relatively close proximity of the two kinetochore clusters. The integrated signal was corrected for background fluorescence by subtracting the peripheral pixels from a concentric 8 × 8 box. To avoid contamination of the background by the neighboring kinetochore cluster, we used the median of these pixels instead of the mean value. For anaphase cells, where each cluster appears as a distinct diffraction-limited spot, we fit the intensity distribution with a 2-D Gaussian to define a mask for the intensity distribution. Pixels within this mask were used to calculate signal intensity. The same procedure was applied to images in GFP, mCherry, and FRET channel to calculate the corresponding fluorescence for each selected kinetochore cluster.

### Fluorescence Lifetime Imaging

Fluorescent protein excitation was achieved using a 940 nm wavelength, 100 fs pulsed Ti:Sapphire laser beam with a 79.5 MHz repetition rate (Mai-Tai, Spectraphysics) focused in the specimen plane with a 40×, 1.25 NA water immersion objective (Nikon, excitation power in the focal plane = 18 mW). A 44 × 44 *μ*m region in a manually selected focal plane was scanned using a dual-channel confocal scanner (DCS-120, Becker & Hickl, pixel size = 172 nm). GFP fluorescence was filtered with a 510/42 emission filter and Time Correlated Single Photon Counting was conducted with the SPC-120 system (Becker & Hickl). The fluorescence decay observed for each strain was fit with either single or double exponential decay models convolved with the instrument response function (obtained by measuring Second Harmonic Generation from urea crystal at 1000 nm excitation with the same 510/42 emission filter).

## Results and Discussion

### Design of Calibration Strains Using the Ndc80 Complex

**Figure 1:**
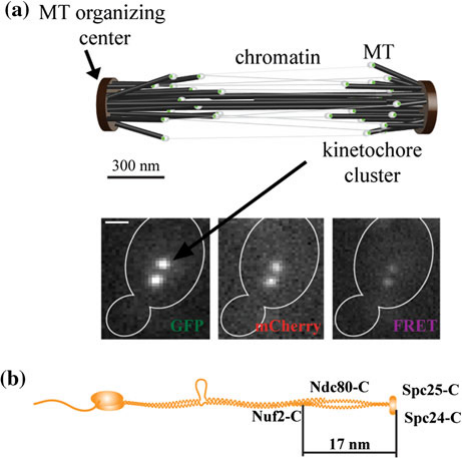
Schematic of the morphology of a budding yeast mitotic spindle in metaphase. (a) Two clusters of sister kinetochores containing 16 kinetochores each organize in each spindle half. The panel displays a cell expressing Ndc80-GFP and Spc25-mCherry in the GFP, mCherry, and FRET channel (scale bar ~2 *μ*m). (b) Architecture of the Ndc80 complex. Known positions of the carboxyl termini of Ndc80 subunits were used to construct three strains with known separations between donor and acceptor fluorophores

To calibrate the epifluorescence microscope for FRET efficiency measurements, we built three distance calibration strains. The budding yeast *Saccharomyces cerevisiae* kinetochore protein complex, Ndc80, was selected as the basis for this calibration. This selection provides key properties for the implementation of our methodology. First, the structure of the Ndc80 complex is known, which allowed us to place the donor and acceptor molecules at distinct separations spanning 10 nm. Second, each kinetochore incorporates a highly specific^9^,^16^,^21^,^30^ (~8 molecules per kinetochore^3^) and stable^16^ number of molecules of the Ndc80 complex. Finally, the morphology of a dividing yeast cell facilitates accurate fluorescence intensity quantification. In a dividing cell, sister kinetochore pairs on 16 chromosomes organize in two clusters, each containing 16 kinetochores (Fig. 1a). When visualized with genetically encoded fluorescent proteins fused to a kinetochore protein, these clusters appear as two distinct fluorescent puncta separated by ~800 nm in metaphase and more than 4 *μ*m in anaphase. Each cluster contains the same number of kinetochores and each kinetochore carries an identical number of Ndc80 molecules, which ensures accurate measurement of cumulative fluorescence.^16^

Each Ndc80 complex molecule consists of four subunits: Ndc80, Nuf2, Spc24, and Spc25, in a 1:1:1:1 stoichiometry and organized in a well-defined structure^32^ (Fig. 1b). Using the known structure of the Ndc80 complex, we generated donor–acceptor pairs with expected separations of 2.4 nm for the Spc24-GFP/Spc25-mCherry strain,^31^ ~5 nm for the Ndc80-GFP/Nuf2-mCherry strain, and >10 nm for Ndc80-GFP/Spc25-mCherry strain. Thus, the donor–acceptor separations in these three strains span the FRET range of 10 nm (assuming the Forster radius of 5 nm for the GFP–mCherry FRET pair^1^) allowing the calibration to cover the entire FRET range.

### Quantification of Sensitized Emission Intensity from Kinetochore Clusters

We quantified the fluorescence intensity from each kinetochore cluster in three channels: acceptor, donor and FRET (see “Materials and Methods”). The measured fluorescence from a kinetochore cluster represents the cumulative fluorescence from 16 kinetochores within the cluster, each kinetochore carrying at least eight FRET pairs (corresponding to 8 Ndc80 complex molecules per kinetochore). Due to the crowding of 16 kinetochores within the cluster, inter-kinetochore FRET can occur. To assess the probability of this FRET process, we examined the spatial distribution of kinetochores from EM tomography data on MT length.^33^ Since each MT is bound by a single kinetochore at its tip, MT length distribution serves as the proxy for kinetochore distribution which cannot be visualized in EM micrographs. The median distance between neighboring MTs is ~40 nm, while the MT length can be approximated by a normal distribution with a mean of 324 nm and standard deviation of 145 nm (Fig. 10b in Winey *et al.* 33). Kinetochores residing at the tips of these microtubules are thus unlikely to be within a 10 nm radius of each other. Furthermore, architecture of purified yeast kinetochore particles also reveals that the Ndc80 complex likely resides in close proximity of the MT lattice along its length.^13^ Therefore, we concluded that the inter-kinetochore FRET is minimal in metaphase. FRET between a donor on an Ndc80 molecule with one or more acceptors on neighboring molecules (or *vice versa*) can also occur. As discussed later, this FRET process can be independently measured in heterozygous diploid yeast strains. This measurement is necessary for understanding the architecture of macromolecular machines carrying multiple copies of the same protein.

The fluorescence recorded in the FRET channel includes sensitized emission, which is the acceptor fluorescence due to FRET, and two major sources of contamination.^25^ These include donor bleed-through fluorescence, which is the donor fluorescence that is detected in the acceptor emission window, and acceptor fluorescence due to excitation of the acceptor fluorophores at the donor excitation wavelengths. To quantify these two sources of contamination in the FRET channel, we constructed additional strains expressing only GFP-labeled or mCherry-labeled Ndc80 complex subunits. By imaging these strains under experimental imaging conditions, we determined the donor bleed-through to be 5.8 ± 0.01% of the fluorescence intensity measured in the GFP channel (Fig. S1). Similarly, mCherry cross-excitation was found to be 6.1 ± 0.02% of the fluorescence measured in the mCherry channel. GFP fluorescence at the mCherry excitation wavelength in the FRET channel was negligible for our imaging conditions.

The estimated GFP bleed-through signal and mCherry cross-excitation signal were both subtracted from the FRET signal to obtain sensitized emission. The calculated sensitized emission was then normalized to obtain the ‘Proximity Ratio’ by dividing sensitized emission by the sum of donor bleed-through and acceptor cross-excitation:

$$ {\text{Proximity ratio}} = \frac{{{\text{Sesitized}}\;{\text{emission}}}}{{{\text{GFP }}\;{\text{bleed-through}} + {\text{mCherry}}\;{\text{cross-excitation}}}} $$

The proximity ratio can be related to the previously established FRET metric^25^ as (FRET_R_—1). These FRET metrics must be used with care when applied to quantify FRET between two proteins with unequal numbers and if the apparent brightness of the donor and acceptor fluorophore is unequal. The use of intensities in the normalization means that the proximity ratio value is influenced by the labeling scheme. The proximity ratio will change if this labeling scheme is reversed, even though the number of FRET pairs and the average donor–acceptor separation remains unchanged. This is not a concern in the calibration strains due to the 1:1 stoichiometry of the Ndc80 subunits. Furthermore, the proximity ratio measured also represents the sensitized emission per FRET pair. This is because each Ndc80 complex molecule is carries one GFP and one mCherry molecule due to its subunit stoichiometry.

### Sensitized Emission is Accurately Measured over a Wide Range of Donor–Acceptor Separations

**Figure 2:**
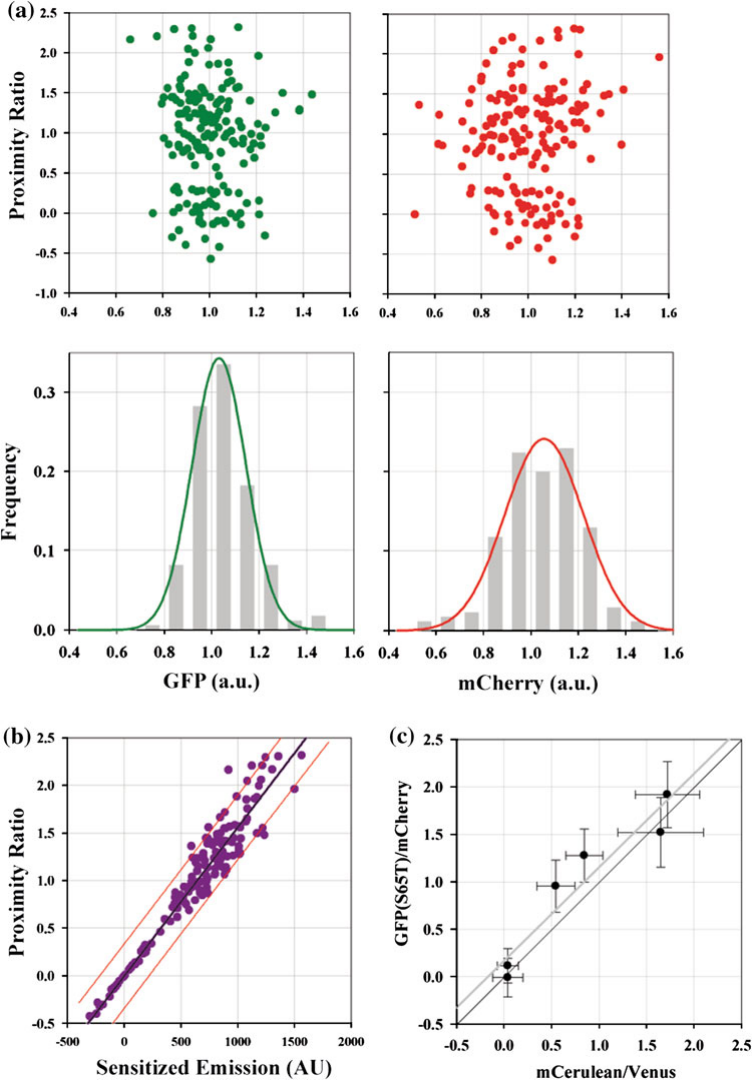
Quantification of sensitized emission from the calibration strains. (a) Scatter plot of the proximity ratio measured for each kinetochore cluster against the corresponding GFP (green) and mCherry (red) intensity values normalized using the average intensity of the dataset. Histograms of GFP and mCherry fluorescence (normalized as above). The green and red lines are fit to the histograms with the normal distribution. (b) Proximity ratios calculated for three strains plotted against the sensitized emission intensity. Black line displays linear regression of the data and red lines display 95% confidence intervals (*N* = 170, *R* ^2^ = 0.97). (c) Proximity ratios measured with the GFP(S65T)–mCherry FRET pair plotted against the values measured with mCerulean–Venus as FRET pair at equivalent positions. Gray line displays linear regression, while the black line passing through the origin displays the expected relationship. The offset between the two lines is due to a systematic under-correction of Venus cross-excitation in our experiment

To determine the accuracy of the intensity measurements and sensitized emission calculation, we plotted the proximity ratios measured in all three strains in metaphase and anaphase as a function of corresponding GFP, mCherry, and sensitized emission intensity (Fig. 2a, GFP and mCherry signals are normalized using the respective cumulative average values). These plots reveal three key points. First, the cell to cell variation in the mCherry signal from kinetochore clusters is significantly larger than the variation in the GFP signal (Fig. 2a lower panel, *σ*_GFP_ = 0.114, *σ*_mCherry_ = 0.166, *p*-value <10^−11^ using one-tailed *F*-test for equal variance as measured in the Ndc80-GFP/Spc25-mCherry strain wherein FRET is minimal). Since each kinetochore cluster contains a stereotypical number of molecules, this variation likely results from variable maturation of the mCherry fluorophore. mCherry maturation relative to GFP has been shown to be inefficient using Fluorescence Cross-Correlation Spectroscopy, which revealed that only 45% of tandem EGFP-mCherry molecules contained an active mCherry.^26^ We found that the sensitized emission was positively correlated with the measured mCherry signal (correlation coefficient = 0.246, *p*-value = 0.001). This positive correlation is expected: a larger number of fluorescent acceptor molecules within the kinetochore yields a larger sensitized emission. We did not find significant correlation between the GFP fluorescence and the proximity ratio (correlation coefficient = −0.047, *p*-value = 0.07), confirming that GFP maturation is uniform from cell to cell. As expected, the proximity ratio increased linearly with the sensitized emission (Fig. 2b). This plot demonstrates that we can accurately quantify FRET over the range of expected donor–acceptor separations used for calibration.

EGFP variants such as mCerulean and Venus constitute a commonly used FRET pair. Therefore, we compared the performance of mCerulean–Venus FRET pair with that of GFP–mCherry. We measured nearly identical proximity ratios for the Ndc80 complex measurements using this FRET pair (Fig. 2c). Due to the superior photostability of GFP and mCherry relative to both Cerulean and Venus,^29^ as well as better spectral separation, the GFP–mCherry FRET pair is better suited for accurate and dynamic FRET measurements.

### Calibration Between FRET Efficiency and Sensitized Emission

**Figure 3:**
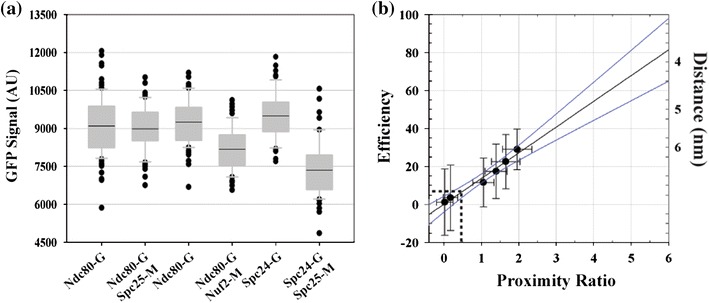
FRET efficiency measurements. (a) Quantification of donor intensities in donor only and FRET strains (only metaphase data is displayed). (b) Plot of FRET efficiency against the corresponding proximity ratio from the three calibration strains (mean ± SD displayed. Standard deviation for the FRET efficiency values was calculated using the error propagation equation for the ratio of two sample means.^5^ Black line displays linear regression (slope = 0.1355, *y*_0_ = 0.002, *R* ^2^ = 0.98) and blue lines display the 95% confidence intervals. Metaphase and anaphase measurements from the three strains are plotted separately. The distance scale on the right displays the donor–acceptor separation calculated using the Förster equation

To deduce donor–acceptor separations from the sensitized emission measurements, the proximity ratio must be linked to the corresponding FRET efficiency. Therefore, we determined the FRET efficiency by measuring donor quenching in each calibration strain. The stereotypical number of Ndc80 complex molecules within a kinetochore cluster in each yeast cell allowed the accurate quantitation of donor quenching due to FRET. We measured the donor fluorescence without any FRET in strains expressing only GFP under identical excitation intensity (Fig. 3a). The ratio of the GFP signal measured in each of the FRET strains with the corresponding GFP signal in the GFP-only strains revealed the FRET efficiency. Next, we examined the dependence between FRET efficiency and the proximity ratio (Fig. 3b). As expected, the two are directly proportional as revealed by linear regression (slope = 0.1355, *y*_0_ = 0.002, *R* ^2^ = 0.98). We plotted FRET measurements from metaphase and anaphase kinetochore clusters separately, as FRET was found to be systematically higher in anaphase. This increase is likely due to a combination of possible structural and architectural changes within the kinetochore in anaphase, reduced inter-kinetochore FRET due to significantly denser spatial distribution of kinetochores,^16^ and continual maturation of mCherry molecules over time.

The linear dependence between FRET efficiency and the proximity ratio serves as a calibration between the two. Proximity ratio is easy to measure under cell biological conditions with high spatiotemporal resolution. The calibration above overcomes the main drawback of sensitized emission as a FRET metric: the dependence of fluorescence intensity on experimental parameters. It also allows the calculation of average donor–acceptor separation from the FRET efficiency. The high protein density within macromolecular machines can allow multiple FRET processes to occur that are difficulty separate. The FRET quantification presented above likely includes FRET from more than one FRET process. As indicated by the linear dependence between the proximity ratio and FRET efficiency, the calibration is insensitive to this complexity. This is a key advantage of our intensity-based FRET measurements.

### Computation of Donor–Acceptor Separations from FRET Efficiency

The measured FRET efficiency can be used to calculate donor–acceptor separations from the Förster equation:

$$ E = \frac{1}{{1 + (R/R_{0} )^{6} }} $$

where *E* is the efficiency, *R* is the donor acceptor separation, and *R*_0_ is the Förster radius. The accuracy of the donor–acceptor separation computed using this equation depends on whether the rotation of the donor or acceptor molecule is isotropic (or the orientation factor *κ* ^2^ = 0.66). The likelihood of steric hindrance to relative rotation of donor and acceptor molecules can be considerable in the crowded environment of macromolecular machines, even with the use of flexible linkers between the protein labeled and the fluorescent protein. However, the non-linear dependence between FRET efficiency and *R*_0_ mitigates the potential errors arising due to anisotropic fluorophore rotation: a 50% error in the estimate of the orientation factor leads to a 20% inaccuracy in the calculated distance.^7^ Nonetheless, verification of isotropic fluorophore rotation using fluorescence polarization quantification^23^ is necessary for accurate distance calculation.

To assess the donor–acceptor separation predicted by the FRET efficiency measurements, we calculated the separation between Spc24-C and Spc25-C using the Förster equation and an *R*_0_ value of 5 nm for the GFP–mCherry FRET pair. The NMR structure of the globular domain of the Spc24-Spc25 dimer reveals that the distance between the carboxyl termini of the two proteins is 2.4 nm.^31^ However, the separation calculated from FRET efficiency is 6.4 nm. The discrepancy in the calculated distance and predicted distance likely arises out of a combination of three possible reasons: (1) inefficient mCherry maturation, (2) significant size of the fluorescent proteins and the linker used to fuse them to the kinetochore protein adding to the separation between labeled domains, and (3) steric hindrance within the kinetochore limiting isotropic fluorophore rotation. Although the latter two factors will lead to an under-estimation of the FRET efficiency, they do not affect the calibration. This is because these factors affect both FRET efficiency and sensitized emission to the same degree.

The possibility of interaction between one donor and multiple acceptors is significant within the crowded environs of the macromolecular machine. In the calibration strains, for example, one Spc24-GFP molecule may be able to interact not just with the Spc25-mCherry within the dimer, but also with the Spc25-mCherry in the adjacent dimer. As discussed later, heterozygous diploid strains can be used to separately measure the latter component. Since the two FRET processes are expected to be independent, and the FRET efficiency within the dimer can then be estimated using a simple subtraction. Additionally, distribution of donor–acceptor separations for the same FRET process, which is unavailable in the average FRET efficiency measurements, is also a key parameter. Characterization of the distribution of donor–acceptor separations is a challenging proposition for any FRET quantification method. In most cases, it requires additional measurements or information about the structure being studied.

Along with the size of the donor and acceptor molecules and steric hindrance, inefficient mCherry maturation must be explicitly considered if absolute donor–acceptor separation is to be accurately measured. If mCherry maturation efficiency is significantly lower than that of GFP, then each kinetochore cluster will contain two GFP populations: GFP donors with and without a mCherry acceptor. Our intensity based method cannot resolve these two populations. Instead, the calibration links the *average* FRET efficiency to the total number of donor and acceptor molecules *via* the proximity ratio normalization. Thus, inefficient mCherry maturation will lead to a systematic under-reporting of the true FRET efficiency and hence over-estimation of the actual donor–acceptor separations.

### Fluorescence Lifetime Measurement to Quantify the Relative Maturation Efficiencies of GFP and mCherry

To test whether significantly lower mCherry maturation leads to two populations of donors in the calibration strains, we used FLIM. FLIM determines FRET efficiency by quantifying the decrease in the lifetime of the donor fluorophore due to FRET to a nearby acceptor. If two donor populations are present, e.g. donors with and without a nearby acceptor, then life-time corresponding to each donor population can also be separated. Using FLIM we first confirmed that the lifetime of GFP(S65T) measured in donor-only strains is consistent with its published values^30^ (Table 1, Fig. S2). We also found that the fluorescence decay measured in the two FRET strains: Ndc80-GFP, Nuf2-mCherry and Spc24-GFP, Spc25-mCherry, consisted of two components. A two-component fit to this decay revealed that the fluorescence lifetime of >70% of the GFP donors was unchanged. These molecules did not encounter an acceptor even though Ndc80 complex structure ensures that every GFP-labeled subunit is accompanied by an acceptor mCherry-labeled subunit within 10 nm in these strains. Therefore, we concluded that a large fraction of mCherry molecules do not form fluorophores, generating the donor population with unchanged fluorescence decay kinetics. The fluorescence lifetime of the remaining GFP molecules was significantly reduced due to FRET (Table 1). Thus, FLIM measurements reveal that poor mCherry maturation is the major factor responsible for the low FRET efficiencies measured by donor-quenching quantitation.

**Table 1:** Fluorescence lifetime measurements (lifetime: mean ± SD)

Strain	Amplitude A1	Life-time 1 (ns)	Amplitude A2	Life-time 2 (ns)	Reduced *χ* ^2^	DA/*D*_total_	*E*
Ndc80-GFP	745.5	2.24 ± 0.04	–	–	0.9	–	–
Spc24-GFP	494.3	2.28 ± 0.06	–	–	1.2	–	–
Ndc80-GFP/Spc25-mCherry	892.8	2.3 ± 0.04	–	–	1.2	–	0
Ndc80-GFP/Nuf2-mCherry	828.4	2.24	242	0.66 ± 0.1	1.1	0.23	70.6%
Spc24-GFP/Spc25-mCherry	532.1	2.28	239.4	0.61 ± 0.08	1.1	0.31	73.1%

DA/*D*_total_ was calculated as (A2/(A1 + A2)). *E* is the FRET efficiency calculated form shorter lifetime (1 − *τ*_2_/*τ*_1_)

It should be noted that donor quenching measurements reveal a larger difference between the FRET efficiency for the Spc24-GFP, Spc25-mCherry strain and the Ndc80-GFP, Nuf2-mCherry strain (29 vs. 17% in anaphase and 22.6 and 9.6% in metaphase respectively). The much smaller difference in the two FRET efficiencies seen in the FLIM analysis may be due to the over-simplification of multiple FRET processes by the two-component fit used in the FLIM analysis.

### A Strategy for Measuring the Proximity Between Adjacent Molecules of the Same Protein

Macromolecular machines usually incorporate multiple copies of the same protein within their structure. Therefore, a key aspect of macromolecular architecture is the separation between adjacent molecules of the same protein. This inter-molecular FRET can be expected to contribute to the detected FRET in our Ndc80 complex strain. This is because each kinetochore contains ~8 Ndc80 complex molecules, each of which binds along the axis of a protofilament of the MT. Since there are 13 protofilaments within the MT, two adjacent Ndc80 molecules can be expected to be 6.15 nm apart.^2^ A strategy is needed to separate FRET occurring between donor and acceptor on the same molecule from the FRET that occurs between the donor on one molecule and the acceptor on a neighbor.

**Figure 4:**
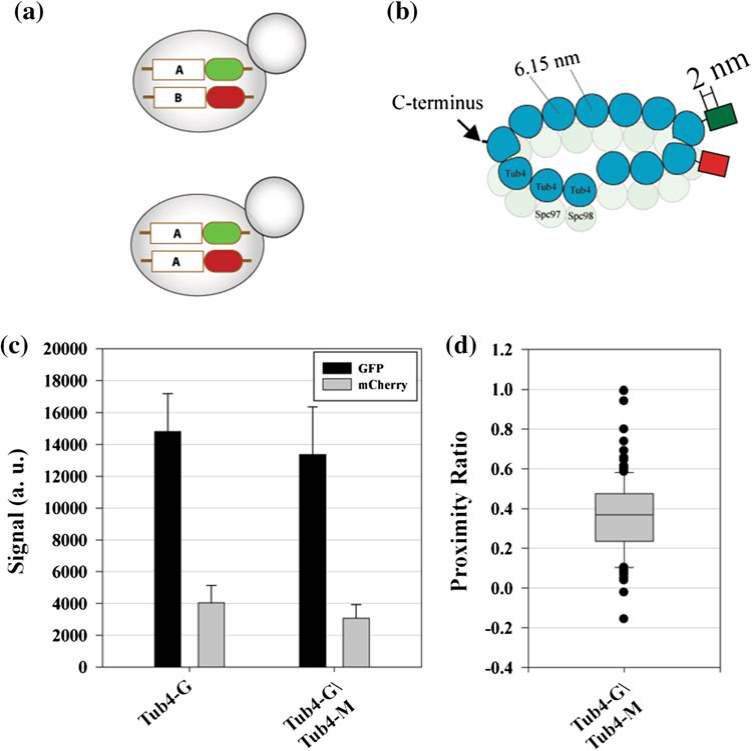
Strategy for measuring FRET between adjacent molecules of the same protein. (a) Haploid and diploid yeast strains can reveal the proximity between adjacent molecules of two proteins or the same protein respectively. (b) The known organization of the γ-TuRC. (c) Fluorescence intensity quantitation from MT organizing centers in a haploid strain expressing either Tub4-GFP or Tub4-mCherry alone and in the heterozygous diploid strain expressing Tub4-GFP/Tub4-mCherry. (d) Box plot of the proximity ratio measurements for the Tub4-GFP/Tub4-mCherry strain (The line within the box displays the median. Edges of the box represent 25th and 75th percentile and the whiskers extend to the extreme values. Outliers are displayed individually as circles)

Measurement of inter-molecular FRET can be easily accomplished using heterozygous diploid budding yeast strains. Diploid yeast cells are constructed by mating budding yeast cells with opposite mating types and carrying the same protein labeled with GFP and mCherry. Such cells will assemble macromolecular complexes by randomly incorporating GFP- and mCherry labeled molecules (Fig. 4a). FRET measured in these cells will reveal the proximity between adjacent molecules of the same protein. To demonstrate this approach, we used the known structure of the γ-tubulin ring complex (γ-TuRC)^18^ (γ-TuRC, Fig. 4b). *in vitro*, Tub4 molecules organize into a helical structure with a 13-fold symmetry. The diameter of this structure is 25 nm matching closely with the diameter of a MT.^18^ For the purpose of this study, we approximated this Tub4 arrangement with the MT lattice structure so that adjacent Tub4 molecules can be expected to be ~6.15 nm apart within the γ-TuRC.

A dividing yeast cell contains two MT Organizing Centers (MTOCs), each one of which nucleates ~20 MT.^33^ Therefore, like the kinetochore, each MTOC incorporates a well-defined number of Tub4 molecules.^12^ The MTOC becomes twice as large in size in diploid cells, and likely contains twice the number of Tub4 molecules. If a diploid strain expresses both Tub4-GFP and Tub4-mCherry, then each MTOC should ideally incorporate nearly equal numbers of Tub4-GFP and Tub4-mCherry. Furthermore, the GFP and mCherry fluorescence measured in these strains should equal the measurements from haploid strains expressing either Tub4-GFP alone or Tub4-mCherry alone.

We quantified FRET at the carboxyl termini of Tub4 in a diploid strain expressing Tub4-GFP/Tub4-mCherry. Due to the size of the MT organizing center in budding yeast, fluorescent protein fusions of Tub4 appear as two well-separated diffraction-limited spots in dividing budding yeast cells. Measurement of Tub4-GFP and Tub4-mCherry fluorescence from each MTOC showed that the two species of the Tub4 molecule are recruited in approximately equal numbers to ensure that each Tub4 ring included the maximum possible number of FRET pairs (Fig. 4b). We measured a proximity ratio of 0.43 ± 0.2 (*N* = 132). This proximity ratio translates into a 5.9% FRET efficiency or an average separation of ~8 nm between adjacent Tub4 molecules (assuming *R*_0_ = 5 nm). If only the maturation efficiency of mCherry molecules relative to GFP is only 30% as suggested by the FLIM measurements, then the true FRET efficiency can be expected to be 3-fold higher (~18%) or a donor–acceptor separation of 6.44 nm. This separation is in excellent agreement with the expected average separation of 6.15 nm between adjacent Tub4 molecules.

Although MTOCs in heterozygous diploid cells are expected to incorporate equal numbers of GFP and mCherry-labeled molecules that total up to twice as many as haploid strains. However, the Tub4-GFP signal measured in the diploid strain is ~90% smaller than the Tub4-GFP signal from a haploid strain. Similarly, the mCherry signal is also ~76% smaller (Fig. 4c). It is possible that the reduced protein counts indicated by the fluorescence measurements reflect a reduction in the cellular protein level due to biological differences in the structure of the MTOC. Alternatively, the faster growth rate of diploid cells may lower the maturation of fluorescent proteins leading to lower fluorescence than expected.

### Discrepancy Between FRET Pair Number and Donor/Acceptor Numbers Requires a Correction for Accurate FRET Efficiency Calculation

The use of the proximity ratio as the measured variable in our calibration necessitates an additional consideration to ensure accurate FRET efficiency deduction in heterozygous diploid strains. The proximity ratio normalizes the measured sensitized emission relative to the *total* number of donor and acceptor molecules. In the calibration strains, the proximity ratio also represents the average sensitized emission per FRET pair. This is because the subunit stoichiometry within the Ndc80 complex ensures an equal number of donor and acceptor molecules in these strains (neglecting the poor maturation efficiency of mCherry). As a result, the number of FRET pairs is either equal to or directly proportional to the total number of donor and acceptor molecules. If this condition is met in an experimental strain, then the measured proximity ratio can be converted into the average FRET efficiency using our calibration.

**Figure 5:**
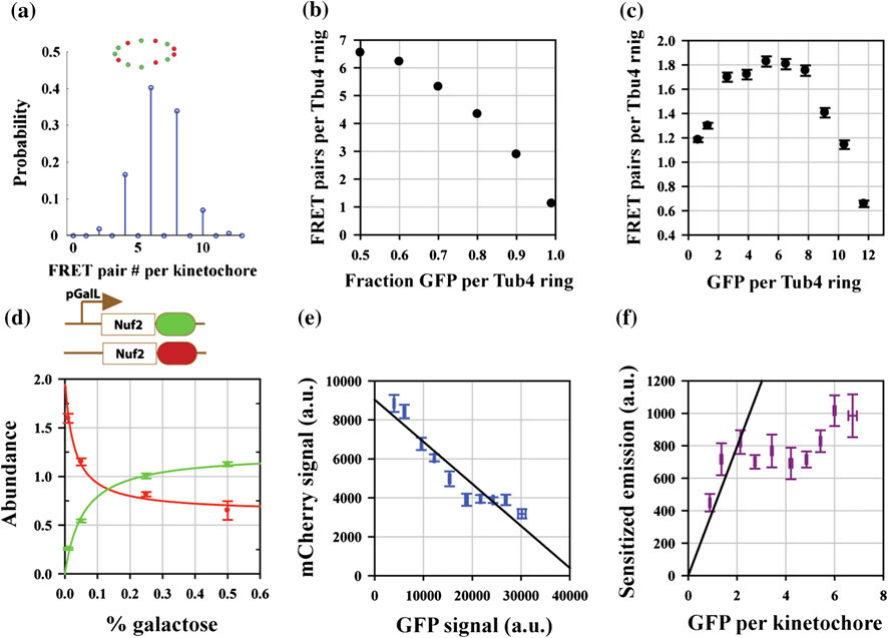
Optimal relative abundance of GFP and mCherry-labeled molecules for maximal FRET. (a) 13 Tub4 positions within the ring structure were randomly assigned GFP or mCherry designation. The resultant number of FRET pairs was determined as the number of GFP donor with a mCherry acceptor within 10 nm, allowing for multiple acceptors per donor. Lower panel displays the probability distribution resulting from 10,000 simulations. (b) Biased incorporation of the GFP donor leads to a decrease in the average number of FRET pairs per γ-TuRC. (c) Poor mCherry maturation leads to the attainment of maximal FRET for lower abundance of Tub4-GFP than predicted by simulations in b (data points represent mean ± SE). (d) Systematic variation of kinetochore fluorescence from Nuf2-GFP and Nuf2-mCherry (normalized using the haploid GFP and mCherry signals as in Fig. 2a, hence the maximum value of 2 on the *Y*-axis) at different galactose concentrations in the media. Increasing concentrations of galactose in the media result in stronger incorporation of Nuf2-GFP in kinetochore clusters (mean ± SE). The observed increase in Nuf2-GFP abundance can be modeled by one site saturation by a ligand (*y*_GFP_ = 2/(*a* + *b*/*x*), *R* ^2^ = 0.98). The decrease in Nuf2-mCherry as a result of Nuf2-GFP over-expression was modeled as a hyperbolic decay (*y*_mCherry_ = 2 − 2/(*a* + *b*/*x*), *R* ^2^ = 0.99). The model assumes that intracellular Nuf2-GFP concentration is proportional to the galactose concentration in the media. (e) The data in (c) re-plotted to display the dependence between Nuf2-mCherry and Nuf2-GFP signals. The black line represents the linear regression (*y*_0_ = 9039 ± 489; *x*_0_ ~42000, *R* ^2^ = 0.9). (f) Plot of total sensitized emission per kinetochore function against the number Nuf2-GFP per kinetochore (mean ± SE). Black line represent linear regression for the first three data points and the origin (*R* ^2^ = 0.9). The number of Nuf2-GFP molecules per kinetochore was calculated by dividing the GFP signal from the cluster with the brightness of a single GFP under the imaging conditions. The latter value was predicted from the total signal in haploid strains expressing Ndc80-GFP only and assuming that there 8 Ndc80 complex molecules per kinetochore

If the structure being studied preferentially recruits either donor or acceptor labeled molecules, then the FRET pair number will be smaller than the total number of donor and acceptor molecules. Therefore, we examined the effect of preferential recruitment of either donor or acceptor-labeled molecules on proximity ratio using Monte Carlo simulations. The simulations were based on a simplified structure of the Tub4 ring: 13 molecules organized in a circular ring of 25 nm diameter. Each simulation assigned the designation of GFP or mCherry randomly to each position, and then calculated the total number of FRET pairs as the number of GFP molecules with mCherry neighbors located within 10 nm allowing for multiple acceptors for each donor (Fig. 5a, inset). The probability distribution of the number of FRET pairs per Tub4 ring shows that the average number of FRET pairs per ring (6–7) is also equal to the number of donor and acceptor labeled molecules per ring (Fig. 5a).

Next, we introduced a bias in the incorporation of one fluorophore over the other, keeping the Tub4 number per ring constant. The number of FRET pairs per γ-TuRC decreases with increasing bias in the recruitment of donor-labeled molecules, and this decrease is approximately linear with the bias (Fig. 5b). Sensitized emission, and hence the proximity ratio, will also decrease, since it is directly proportional to the number of FRET pairs. Therefore, the measured proximity ratio will yield a lower FRET efficiency than its actual value, even though the donor–acceptor separation is unchanged. A correction to the measured proximity ratio is necessary, and the plot in Fig. 5b shows that this correction can be assumed to be proportional to the degree of bias. This exercise demonstrates that maximal FRET is obtained only when equal numbers of donor and acceptor molecules are present in the structure being measured. If this condition is not met, a correction that depends on the relative donor–acceptor abundance must be applied to estimate maximal FRET.

It should be noted that the above discussion assumes 100% maturation of donor and acceptor molecules. The poor mCherry maturation revealed by FLIM data suggests that even with equal recruitment of GFP and mCherry-labeled protein, fluorescent GFP molecules will outnumber fluorescent mCherry molecules. As a result, maximal FRET can be expected to occur for a lower than optimal abundance of GFP-labeled protein. To test this hypothesis, we ran Monte Carlo simulations to include 25% maturation efficiency for mCherry. Relative abundance of GFP and mCherry (total numbers) per ring was varied systematically as above, and the number of FRET pairs per Tub4 ring was determined in each experiment. As expected, maximal FRET occurs for 3–4 Tub4-GFP per Tub4 ring instead of 6.5 Tub4-GFP (Fig. 5c).

### Low mCherry Maturation Efficiency Affects the Optimal Abundance of GFP and mCherry-Labeled Proteins

To experimentally determine the relationship between FRET and relative GFP–mCherry abundance, we constructed a heterozygous strain involving Nuf2, an Ndc80 complex subunit. Nuf2 was used as the test as an analogous well-behaved strain involving Tub4 could not be obtained. The expression of Nuf2-GFP was controlled by the galactose promoter^14^ pGalL, while Nuf2-mCherry was constitutively expressed by the endogenous promoter (Fig. 5d, inset). This strain was grown in media containing different galactose concentrations, and FRET from anaphase kinetochore clusters was measured (*N* > 50 for each galactose concentration). Predictably, higher galactose concentrations resulted in higher Nuf2-GFP expression, stronger recruitment of Nuf2-GFP in kinetochores, and a corresponding decrease in the recruitment of Nuf2-mCherry (Fig. 5c). Next, we binned FRET measurements for all the galactose concentrations according to the number of GFP labeled molecules per kinetochore calculated from the GFP fluorescence measured for each cluster. Although the total number of Nuf2 molecules per kinetochore remained almost constant for lower expression values of Nuf2-GFP, it increased for high expression of Nuf2-GFP (Fig. 5e, last four data points).

We examined the dependence of sensitized emission on the Nuf2-GFP number per kinetochore (Fig. 5f). Sensitized emission was used to avoid the fluorescence-dependent proximity ratio normalization. We found that sensitized emission, which can be assumed to be proportional to the number of FRET pairs per kinetochore, increased linearly initially until it reached the maximal value for ~2–3 Nuf2-GFP molecules per kinetochore. In a manner similar to the Tub4 simulations, the sensitized emission per kinetochore cluster leveled off beyond this number. Thus, the low maturation efficiency of mCherry makes correction based on the relative abundance of GFP and mCherry difficult.

Although we expected the sensitized emission to decrease for high numbers of Nuf2-GFP per kinetochore, it increased for the highest Nuf2-GFP concentrations. This increase is likely because of the increase in the cumulative number of Nuf2 molecules per kinetochore (see the last four points in Fig. 5e). Although the Ndc80 complex is recruited to the kinetochore *via* the Mtw1 complex, an additional linkage through the protein Cnn1 is also available, but not used in metaphase.^6^ We speculate that the additional Nuf2 or Ndc80 complex molecules may be recruited *via* this mechanism changing their architecture at high, non-physiological Nuf2 expression levels.

## Conclusions

We have established a simple scheme for calculating FRET efficiency from sensitized emission intensity measurements. This scheme is based on accurate quantitation of sensitized emission in a set of calibration strains, and then using a calibration between sensitized emission and FRET efficiency to deduce the donor–acceptor separation in experimental samples. We also developed this scheme to measure the average proximity between two molecules of the same protein, and demonstrated its use by measuring the distance between adjacent molecules of γ-tubulin in the γ-TuRC.

Our method is ideally suited for the study of discretely localized macromolecular machines or assemblies within a cell, which present a unique challenge to conventional techniques of FRET efficiency measurement. Biochemical purification or reconstitution of such machines is usually difficult, making *in vivo* studies necessary. Such machines and assemblies also incorporate a relatively small number of copies of each subunit, which limits the number of photons that can be extracted from fluorescent labels on the subunits before they photobleach. The limited number of photons can limit the accuracy and temporal resolution in FLIM measurements. Our approach alleviates this limitation by detecting sensitized emission fluorescence with a high quantum efficiency CCD cameras commonly used in cell biological research. Such highly sensitive detectors allow accurate fluorescence quantitation over a wide range of fluorophore number with the highest possible spatiotemporal resolution. These advantages will enable the use of our FRET quantification approach under cell biological conditions for both static and dynamic measurements.

Genetically encoded fluorescent proteins are an essential tool in defining the *in vivo* architecture of macromolecular machines. However, they also impose significant limitations on the use of FRET for distance measurements. Our study traces these limitations to two factors: (1) the large size of fluorescent proteins relative to the usable FRET range, and (2) poor maturation efficiency of the mCherry fluorophore. Therefore, FRET-based distance estimation *in vivo* using genetically encoded fluorescent proteins can be a challenging proposition. It should be noted, if these limitations are minimized, then accurate FRET-based determination of macromolecular architecture can be achieved.^25^

Despite the unavoidable limitations associated with the use of genetically encoded fluorophores, our FRET-based method can be highly effective in determining key facets of macromolecular architecture. The main challenge in studying macromolecular architecture is not the structures of constituent proteins. Structural biological methods are available for obtaining molecular structures with atomic resolution, and tremendous progress is being made in solving the structures of cellular proteins and protein complexes. For understanding cell biological organization and function, the important measurement is the relative proximity of protein molecules relative to other copies of the same protein or other proteins. The FRET-based analysis method presented here can be highly effective in obtaining this information. It is especially powerful, if the structures of constituent proteins are already available.

Emerging technologies also promise to overcome the limitations of fluorescent proteins for FRET-based distance measurements *in vivo*. In particular, unnatural, fluorescent amino acids hold great promise, as they allow site-specific labeling of proteins with small fluorophores that are also genetically encoded.^22^ Efforts are also underway to engineer mutant variants of fluorescent proteins with improved spectral and maturation characteristics.^20^ The availability of a well-suited acceptor fluorophore will alleviate the drawbacks imposed by mCherry as the acceptor.

Finally, our FRET quantification scheme can be adapted to any other model systems. It will be especially useful in the characterization of dynamic macromolecular protein assemblies such as the endocytic coat, wherein multiple copies of many different proteins loosely organize in a discretely localized structure. Importantly, the measurements can be carried out without the use of any specialized equipment. In conclusion, this scheme FRET quantification will be useful for studying both static and dynamic architecture of a wide range of cellular machines *in vivo*.

## Electronic supplementary material

Below is the link to the electronic supplementary material.

Supplementary material 1 (DOCX 5483 kb)

## Abbreviations

FRET: Forster Resonance Energy Transfer
FLIM: Fluorescence Lifetime Imaging
MT: Microtubule
γ-TuRC: γ-Tubulin ring complex
