# Supplementary material for: A Sensitized Emission Based Calibration of FRET Efficiency for Probing the Architecture of Macromolecular Machines
Source: Cell Mol Bioeng. 2013 Jul 11;6(4):369–82. doi: 10.1007/s12195-013-0290-y (PMC3843746; doi:10.1007/s12195-013-0290-y)
Supplement: Supplementary file 1 — Supplementary material 1 (DOCX 5483 kb) [file 12195_2013_290_MOESM1_ESM.docx]

**
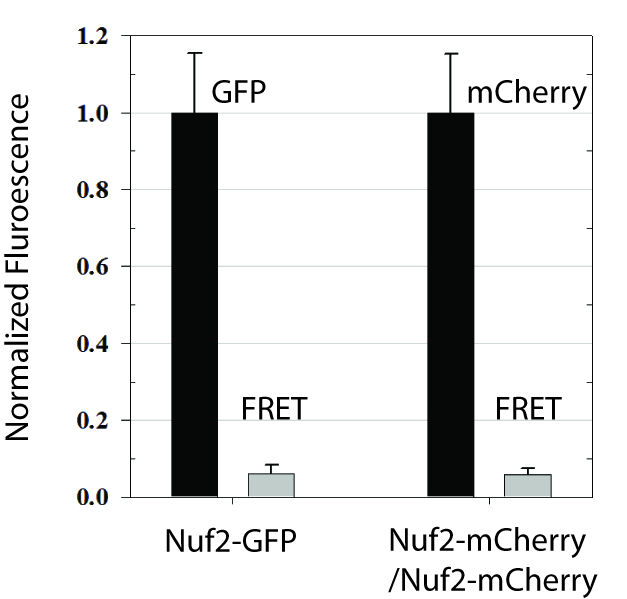
**

**JOGLEKAR FIGURE S1 Empirical determination of GFP bleed-through and mCherry cross-excitation in measured in the FRET channel.**

**
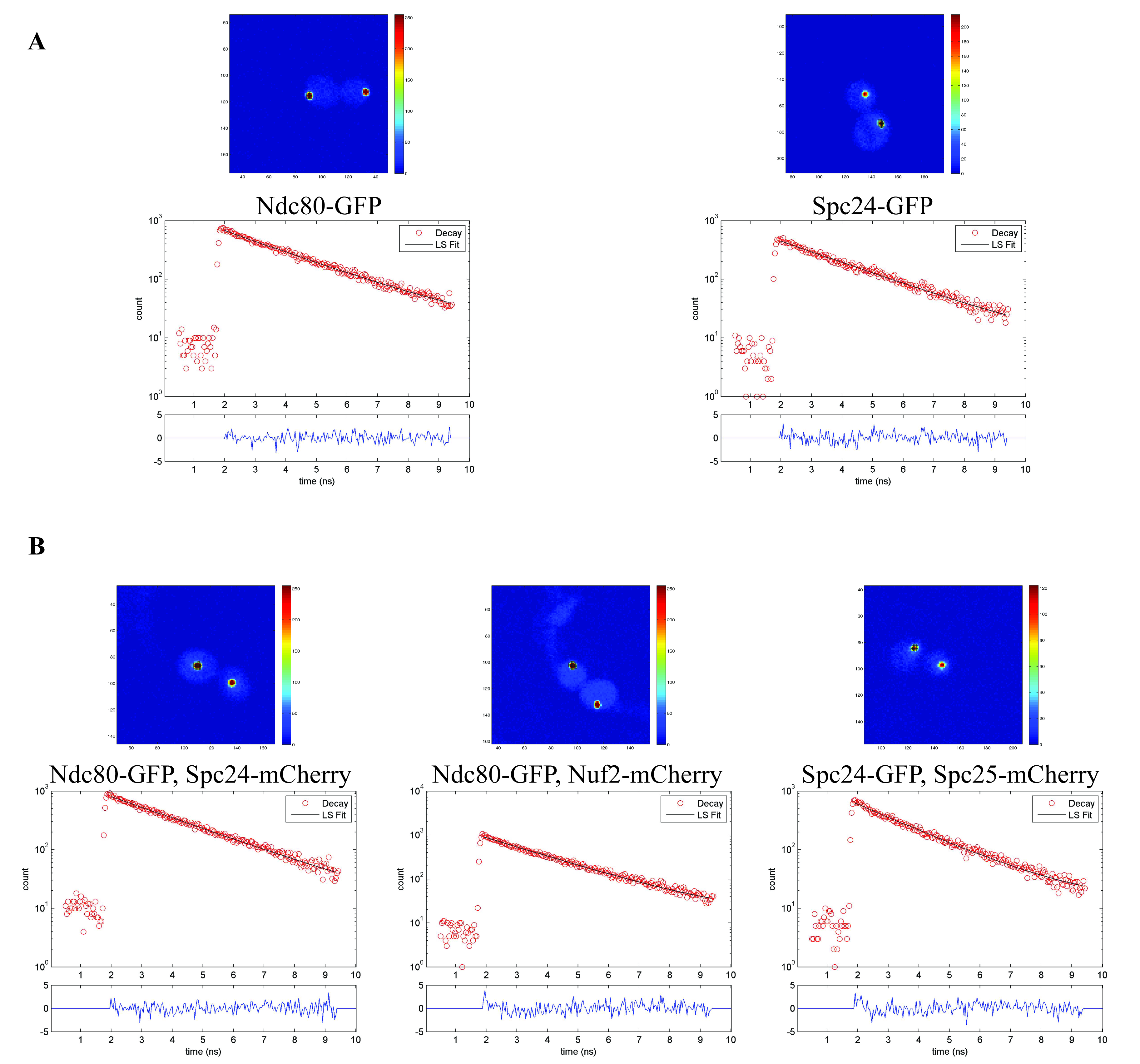
**

**JOGLEKAR FIGURE S2 Fluorescence Lifetime Imaging of donor-only and FRET strains. For each strain, photon counts from 5 cells were combined to obtain the average lifetime.**
